# Parent anion radical formation in coenzyme Q_0_: Breaking ubiquinone family rules

**DOI:** 10.1016/j.csbj.2022.12.011

**Published:** 2022-12-09

**Authors:** J. Ameixa, E. Arthur-Baidoo, J. Pereira-da-Silva, M. Ončák, J.C. Ruivo, M.T. do N. Varella, F. Ferreira da Silva, S. Denifl

**Affiliations:** a Institut für Ionenphysik und Angewandte Physik, Leopold-Franzens Universität Innsbruck, Technikerstraße 25/3, 6020 Innsbruck, Austria; b CEFITEC, Department of Physics, Universidade NOVA de Lisboa, 2829-516 Caparica, Portugal; c Center for Molecular Biosciences (CMBI), Leopold-Franzens Universität Innsbruck, Technikerstraße 25/3, 6020 Innsbruck, Austria; d Institute of Physics, University of São Paulo, Rua do Matão 1731, 05508-090 São Paulo, Brazil

**Keywords:** Ubiquinone, *Para*-benzoquinone, Electron attachment, Electron transfer, Resonance, Dipole-bound anion radical

## Abstract

We report electron attachment (EA) measurements for the parent anion radical formation from coenzyme Q_0_ (CoQ_0_) at low electron energies (<2 eV) along with quantum chemical calculations. CoQ_0_ may be considered a prototype for the electron withdrawing properties of the larger CoQ*_n_* molecules, in particular ubiquinone (CoQ_10_), an electron carrier in aerobic cell respiration. Herein, we show that the mechanisms for the parent anion radical formation of CoQ_0_ and CoQ*_n_* (*n* = 1,2,4) are remarkably distinct. Reported EA data for CoQ_1_, CoQ_2_, CoQ_4_ and *para*-benzoquinone indicated stabilization of the parent anion radicals around 1.2–1.4 eV. In contrast, we observe for the yield of the parent anion radical of CoQ_0_ a sharp peak at ∼ 0 eV, a shoulder at 0.07 eV and a peak around 0.49 eV. Although the mechanisms for the latter feature remain unclear, our calculations suggest that a dipole bound state (DBS) would account for the lower energy signals. Additionally, the isoprenoid side chains in CoQ*_n_* (*n* = 1,2,4) molecules seem to influence the DBS formation for these compounds. In contrast, the side chains enhance the parent anion radical stabilization around 1.4 eV. The absence of parent anion radical formation around 1.4 eV for CoQ_0_ can be attributed to the short auto-ionization lifetimes. The present results shed light on the underappreciated role played by the side chains in the stabilization of the parent anion radical. The isoprenoid tails should be viewed as co-responsible for the electron-accepting properties of ubiquinone, not mere spectators of electron transfer reactions.

## Graphical abstract

**Figure:**
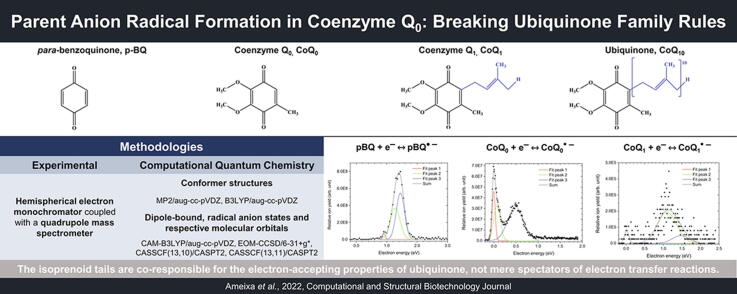

## 1 Introduction

The quinone group is present in electron-withdrawing molecules relevant to biology, medicine and materials sciences. For instance, quinones have been suggested for the treatment of renal disorders [1], diabetes [2], and cancer [3], [4]. Quinone derivatives find applications in flow batteries [5], zinc-organic batteries [6], and also as dopants in organic electronics [7], [8]. Plastoquinone serves as an electron carrier in the electron transport chain associated with the light-dependent reactions of photosynthesis [9], while coenzyme Q_10_ (CoQ_10_), also known as ubiquinone, plays a similar role in aerobic cell respiration [10]. It is generally accepted that the isoprenoid side chain of the coenzyme takes part in intermolecular interactions [11], [12], [13], [14]. The mitochondrial Complex I has an l-shaped structure. Electrons produced by NADH oxidation are transported along the hydrophilic arm through a series of Fe-S clusters. The second arm, embedded in the mitochondrial inner membrane, provides an amphipathic environment composed of charged and hydrophobic ends. The ubiquinone chamber stretches through the boundary between the two arms [11], [12], and recently reported free energy profiles unveiled the interplay between the interactions of the ubiquinone head and tail [14]. Electron transfer takes place as the CoQ_0_ head approaches the closest lying cluster, labelled N2, while diffusion of the isoprenoid tail along the mitochondrial inner membrane carries the electrons to the subsequent stages of aerobic cell respiration. The CoQ_0_ head can form hydrogen bonds with water molecules (micro-hydration) and nearby amino acid residues in the hydrophilic arm, while the tail is responsible for hydrophobic interactions in the membrane, although with some degree of hydration.

The *para*-benzoquinone (*p*BQ) group is the main responsible for the reduction of the quinone acceptors. The unique electron-accepting properties of the *p*BQ molecule arise from the combination of its large electronic affinity (1.86 eV [15], [16]) with efficient mechanisms for the ultrafast internal conversion of the anion radical, which bypass the inverted Marcus regime [17]. Once low-lying excited states are formed, the anion radical undergoes nearly barrierless transitions to the ground state, rendering *p*BQ a very efficient electrophore. The excited states of the *p*BQ anion radical are resonances (unstable against spontaneous emission of the captured electron) known from time-resolved photoelectron spectroscopy (TRPES) [17], [18], photodetachment data [15], [16], and quantum chemistry calculations [17], [19], [20], [21]. In particular, the TRPES studies have unveiled the non-adiabatic dynamics initiated by optical excitation to the ^2^A_u_ shape resonance and the ^2^B_3u_ core-excited resonance. These temporary negative ions (TNIs) decay to the ^2^B_2g_ ground state in a sub-40 fs time scale through a series of conical intersections, thus suppressing the auto-ionization channels [15], [17], [18]. The complex network of conical intersections involving the ^2^B_2g_, ^2^A_u_, ^2^B_3u_, and other excited anion radical states was characterized in detail by recent computational studies [17], [21].

The anion radical states of *p*BQ have also been investigated both experimentally and theoretically through electron attachment (EA) [22], [23], [24], [25], electron transmission [26], [27], [28], and electron scattering [29], [30], [31] studies. The transient nature of the excited anion radical states has also been considered in computational studies employing scattering [32], [33] and modified quantum chemistry methods [34], [35]. The TNIs’ energies obtained from EA to the neutral *p*BQ molecule can be related to those obtained by optically exciting the bound anion radical *p*BQ^•–^, as their differences amount to the electron affinity of the *p*BQ ground state. At sub-electronic excitation energies, the most prominent feature of EA measurements is the formation of the parent anion radical. Quite unusually, it is not observed at ∼ 0 eV, and instead only formed around 1.36–1.56 eV [22], [24], [25]. This uncommon behaviour can be rationalized in light of the TRPES data and supporting calculations. The ^2^B_2g_ ground state lies, vertically, about 1.7 eV below the ground state of the neutral form [17], [21]. This large energy gap suppresses the formation of the parent at low energies, since EA would require vibrational excitation into the continuum. Parent formation at higher energies (≈1.4 eV) should be mediated by the ^2^B_3u_ Feshbach resonance [17], [21], which subsequently undergoes internal conversion to the ^2^B_2g_ ground state through the cascade of conical intersections.

Recently, an EA study to three CoQ_n_ molecules (*n* = 1,2,4; *n* indicates the number of isoprenoid units that form the side chain) was reported [36]. It may be proposed that the smaller members, n⩽4 can be viewed as models for the biologically occurring form observed in mitochondria, CoQ_10_. The CoQ_0_ head is believed to be the electron-accepting site of the CoQ_10_, while the side chain, as mentioned above, accounts for hydrophobic interactions in the ubiquinone binding site of the mitochondrial Complex I [11], [12], [13], [14]. Therefore, the formation of the parent anion radicals by EA to CoQ_1_, CoQ_2_ and CoQ_4_ should share essentially the same features as in *p*BQ. Indeed, the parent anion radical signals were not found at ∼ 0 eV, and instead, anion radical signals were observed around 1.2 eV for the three CoQ*_n_* (*n* = 1,2,4) molecules [36]. The lifetimes of the CoQ*_n_*^•–^ (*n* = 1,2,4) parents may increase with the number of isoprenoid units, pointing out that the length of the isoprenoid tail favours thus parent anion radical stabilization.

From the results outlined above, one would expect the analogous formation of the parent anion radical also for the CoQ_0_ molecule (2,3-dimethoxy-5-methyl-*p*-benzoquinone), having no side chain. This parent anion radical, henceforth indicated as CoQ_0_^•#–^ (the superscript ^#^ indicates that it may be formed in an electronical and/or vibrational excited state), may share essentially the same features, as the latter molecule bridges the gap between *p*BQ and the CoQ*_n_* series (*n* = 1,2,4). That expectation would even be corroborated by TRPES data and supporting calculations [37]. A clear correspondence between the energy of the TNIs of *p*BQ and CoQ_0_ was pointed out, as well as similar internal conversion pathways, although with smaller yields, and thus suggesting that auto-ionization is more favoured in CoQ_0_ compared to *p*BQ. However, in the present gas-phase study, we demonstrate that the formation of CoQ_0_^•#–^ by EA breaks the ubiquinone family rules. Using mass spectrometry, the parent anion radical is only observed below 1 eV, which is in clear contrast with the gas-phase data for *p*BQ [22], [24], [25] and CoQ*_n_* [36] (*n* = 1,2,4). The CoQ_0_ exception is even more intriguing since it is very clear in EA experiments, although not as evident in the TRPES results [37]. Thus, the presently reported EA data suggest that the isoprenoid tails are more involved in the electron withdrawing properties of the CoQ*_n_* molecules than previously thought, in addition to the intermolecular interactions relevant to the bioactivity of ubiquinone.

## 2 Experimental methods

For the present study, we used a hemispherical electron monochromator (HEM) coupled with a quadrupole mass spectrometer (QMS). The setup was described previously in more detail elsewhere [38]. The CoQ_0_ sample (delivered from abcr GmbH, Karlsruhe, Germany, with stated purity of 97 %) was placed as received in an external container attached to a gas line equipped with a precision valve. A stainless-steel capillary (1 mm diameter) guided the sample vapor directly into the interaction chamber of the HEM, where it interacts with the electron beam. The anion radicals formed by electron attachment reactions are extracted by a weak electrostatic field towards the QMS for mass-selection of the parent anion radical of CoQ_0_ (*m*/*z* 182). Other product anions which are formed by dissociative electron attachment were reported in Ref. [39]. In the last step, the yield of parent anion radicals was detected with a channeltron-type secondary electron multiplier operated in single-pulse counting. An electron energy scan for a mass-selected anion radical is measured by recording the number of output pulses per second for each incident electron energy. At an electron current of ∼ 10nA, the HEM produced an electron beam with an energy resolution of about 110 meV as measured from the full-width at half maximum (FWHM) of the ∼ 0 eV resonance for Cl^–^ formation upon electron attachment to CCl_4_ [40]. This reaction was also used to calibrate the electron energy scale. Please note that we have covered the electron energy range from threshold up to about 5 eV, but only sections showing ion signal are provided here.

In addition, we carried out electron attachment studies with *p*B*Q* and CoQ_1_ using the above-described experimental approach and determined the yield of corresponding parent anion radicals as function of the initial electron energy. These additional measurements serve to rule out any discrepancies between the data reported in literature and the present results due to experimental reasons. These data allow a direct comparison of the yields for the three molecules measured under very similar experimental conditions. To give an example, a double focusing mass spectrometer, for which various experimental parameters like electron energy resolution, ion extraction conditions and timescales of ion detection strongly differ to the present experiments, was used to study CoQ_1_ [36]. Here, the *p*BQ and CoQ_1_ samples, as received from Sigma Aldrich, Vienna, Austria (purity ≥ 98 % and ≥ 95 %, respectively), were placed in an external container and their vapor was guided towards the interaction region of the HEM via a stainless-steel capillary. In the *p*BQ measurements, the working pressure was of 2.6 × 10^–8^ mbar, while that for the CoQ_0_ measurements was of 6.0 × 10^–8^ mbar, and for CoQ_1_ it was ∼ 1 × 10^–8^ mbar as measured with a hot-cathode ionization pressure gauge mounted on a flange of the high-vacuum chamber. For each incident electron energy, relative ion yield intensities were subsequently determined by dividing the measured ion yield intensity by the working pressure value. At last, peak positions were determined by fitting multiple Gaussian functions to the measured ion yield intensity, as reported previously [41].

## 3 Theoretical methods

**Fig. 1:**
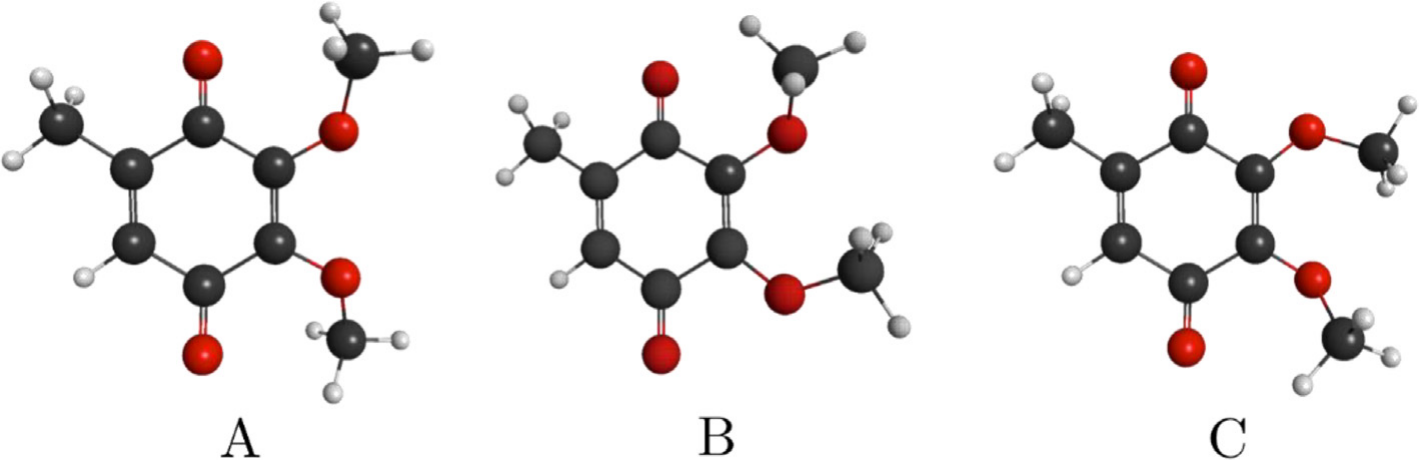
Geometries of the neutral CoQ_0_ conformers labeled A, B and C. The carbon atoms are represented in black, oxygen in red, and hydrogen in gray.

Three stable conformers of CoQ_0_, indicated as A, B and C, were obtained with the conformation search tool built in the Avogadro software [42]. The corresponding geometries are shown in Fig. 1, and were subsequently optimized with either second-order Møller-Plesset perturbation theory (MP2) or density functional theory (DFT) with the aug-cc-pVDZ basis set, using the Gaussian09 package [43]. The A, B and C conformers are labeled in order of increasing energy, although they are nearly degenerate [39]. The conformer B is the most strongly polar, having a permanent dipole moment around 2.2D to 2.5D, depending on the calculation method, while the other structures have dipole moments around 1.7D to 2.0D (conformer C) and 0.7D to 1.1D (conformer A).

**Fig. 2:**
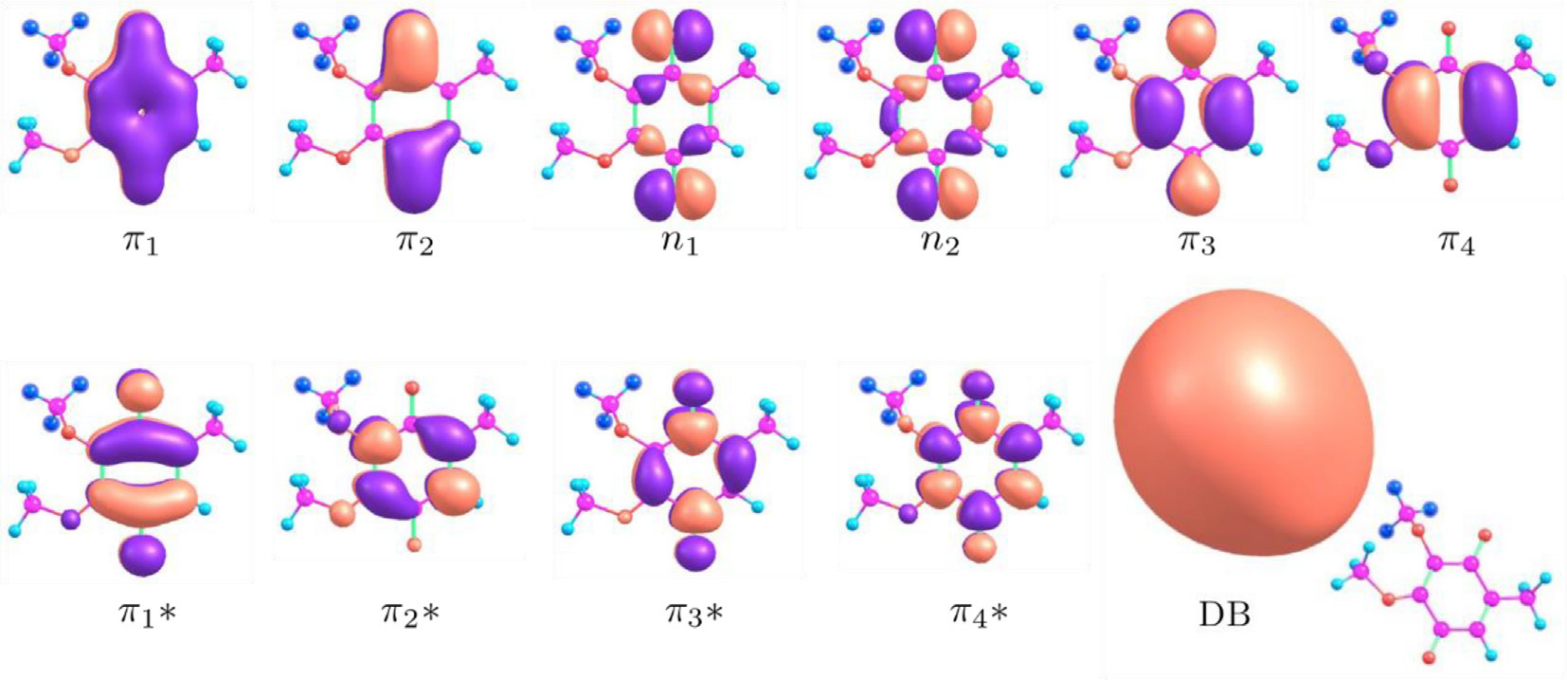
Molecular orbitals included in the active space of the CASSCF calculations for the conformer B. The orbitals in the upper panel are doubly occupied in the reference state (π_4_ is the HOMO of the neutral molecule). In the lower panel, $\pi_{1}^{*}$ is singly occupied in the reference, while $\pi_{2}^{*}$ to $\pi_{4}^{*}$ are unoccupied. After the inclusion of diffuse atomic orbitals (see text), the active space was augmented with the orbital with dipole-bound (DB) character. The diffuse atomic orbitals were placed on the hydrogen atoms (indicated in dark blue color) of the methoxy group at the C3-position of the ring. The other hydrogen atoms are indicated in light blue, carbon in magenta, oxygen in red and double bonds in light green.

Given that CoQ_0_ exhibits a long-lived valence-bound anion radical, its excited anion radical states are resonances and bound-state techniques have proven useful to describe π* states of both *p*BQ^•–^ [17], [20], [44] and CoQ_0_^•–^ [37]. Herein, we investigated the anion radical states of the latter molecule employing different quantum chemistry techniques, namely the complete-active-space self-consistent-field (CASSCF) method with second-order perturbative corrections (CASPT2), employing the geometries of neutral conformers optimized with the MP2/aug-cc-pVDZ method. The CASSCF computations were carried out with the OpenMOLCAS [45] package, using the extended relativistic Atomic Natural Orbital (ANO-L) basis set in the contractions scheme [3s2p1d] for carbon and oxygen atoms and [2s1p] for hydrogen. The active space comprised 13 electrons and 10 orbitals, which is indicated as CASSCF(13,10). The dipole moment of conformer B, in excess of 2 D, would be expected to support a dipole bound state (DBS). To investigate this possibility, we performed an independent calculation with the ANO-L basis set augmented with sets of 7s5p diffuse orbitals placed on the hydrogen atoms of the methoxy group located at the C3-position of the ring (see Fig. 2 and Table S2). A diffuse orbital with DBS character was then included in the CASSCF(13,11) calculation for the anion radical species. The molecular orbitals comprising both active spaces are shown in Fig. 2, and electronic correlation effects were included with second-order perturbation theory (CASPT2). The numerical procedures are given in the Supporting Information (SI). We further investigated the anion radical states with time dependent density functional theory (TDDFT), using the CAM-B3LYP exchange–correlation functional and the aug-cc-pVTZ basis set. Finally, we carried out equation-of-motion coupled-cluster calculations with single and double excitations (EOM-CCSD), employing the 6–31+g* basis set. The TDDFT and EOM-CCSD computations, performed with the Gaussian16 package [46], used standard basis sets, not augmented with very diffuse atomic orbitals, as described above for the CASSCF(13,11) model. Those calculations are therefore not expected to account for a low-lying DBS.

The three conformers have bound anion radical states with π* character, i.e., the respective molecular anion radical is formed by the addition of one electron into the $\pi_{1}^{*}$ orbital. As shown in Table 1, the calculated vertical binding energies range from 1.6 eV to 2.0 eV, depending on the method and the conformer. The inclusion of the diffuse orbital in the CASSCF(13,11)/CASPT2 calculation slightly destabilizes the ground anion radical state with π* character. More importantly and discussed in detail below, it gives rise to a DBS with binding energy of 65 meV, pointing out that CoQ_0_ could attach electrons at thermal energies. The second-order extended multi-configurational quasi-degenerate perturbation theory (XMCQDPT2) calculations reported Bull *et al.* [37] for CoQ_0_ did not account for the DBS. This previous study considered the less polar conformer A at the most stable geometry of the anion radical ground state, without augmenting the aug-cc-pVDZ basis set with extra diffuse orbitals.

**Table 1:** Character and energies of the CoQ_0_ anion radical states, in units of eV, obtained for the conformers A and B. The anion radical state energies are relative to the ground state of the neutral molecules at the geometry of the latter species. Negative and positive energy values indicate bound and transient anion radical states, respectively. The active space of the CASSCF(13,11)/CASPT2 calculation was augmented with a diffuse virtual orbital having dipole-bound (DB) character. Polarizable continuum model (PCM) with water as solvent was used to account for solvent effects. See Table S9 for EOM-CCSD calculations.

	CAM-B3LYP/ aug-cc-pVTZ		PCM-CAM-B3LYP/ aug-cc-pVTZ		CASSCF(13,10)/ CASPT2		CASSCF(13,11)/ CASPT2
Anion radical state character	A	B	A	B	A	B	B
${\left(\pi_{1}^{*}\right)}^{1}$	–1.95	–1.71	–3.92	–3.73	–1.84	–1.72	–1.59
${\left(DB\right)}^{1}$							–0.065
${\left(\pi_{2}^{*}\right)}^{1}$	1.12	1.13	–0.77	–0.57	0.85	1.00	0.80
${\left(\pi_{3}\right)}^{1}{\left(\pi_{1}^{*}\right)}^{2}$	1.21	1.51	–0.60	–0.80	1.15	1.27	1.21
${\left(n_{2}\right)}^{1}{\left(\pi_{1}^{*}\right)}^{2}$	1.10	1.57	–0.80	–0.41	1.11	1.30	1.27
${\left(n_{1}\right)}^{1}{\left(\pi_{1}^{*}\right)}^{2}$	1.03	1.30	–0.88	–0.58	1.16	1.39	1.43
${\left(\pi_{4}\right)}^{1}{\left(\pi_{1}^{*}\right)}^{2}$	1.41	1.65	–0.18	0.03	1.46	1.58	1.68

It is clear from Table 1 that the energy of the anion radical states of CoQ_0_ obtained with TDDFT and CASPT2 methods are in reasonable agreement including the energy of the ${\left(\pi_{3}\right)}^{1}{\left(\pi_{1}^{*}\right)}^{2}$ Feshbach resonance in the conformers A and B, corresponding to the [5]^2^F state assigned by Bull *et al.* [37]. This anion radical state also has a mixed shape/Feshbach character in the CASSCF/CASPT2 computations, while TDDFT indicates two anion radical states with shape ($\pi_{3}^{*}$) and Feshbach (${\left(\pi_{3}\right)}^{1}{\left(\pi_{1}^{*}\right)}^{2}$) characters. Previous studies also pointed out that the character of the ${\left(\pi_{3}\right)}^{1}{\left(\pi_{1}^{*}\right)}^{2}$ resonance is sensitive to the calculation method [33], [37]. In general, the energies computed with the EOM-CCSD/6–31+G* method are overestimated by ∼ 0.6 eV compared to the other calculations (Table S9; at the TD-CAM-B3LYP level, the absolute mean difference in excitation energies when applying 6–31+g* and aug-cc-pVDZ basis sets is below 0.1 eV). Inclusion of the water solvent effects through the polarizable continuum model (PCM) shows that all investigated states are stabilized by about 2 eV compared to the neutral molecule.

## 4 Results and discussion

**Fig. 3:**
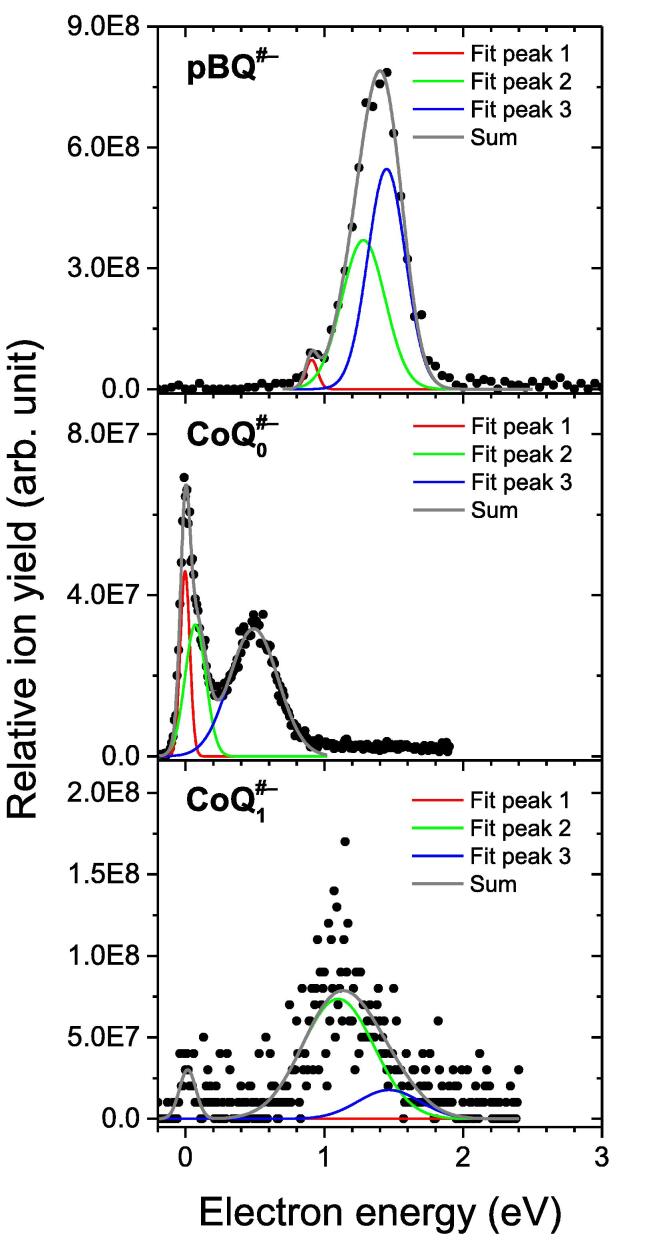
Top panel: Relative ion yield intensity of the molecular anion radical *p*BQ*^•^*^#–^, and multiple Gaussian fitting (*R*^2^ = 0.99), based on three Gaussian functions (fit peak 1, 2, 3) centered at 0.91, 1.28, 1.45 eV, respectively. Middle panel: Relative ion yield intensity of the molecular anion radical CoQ_0_*^•^*^#–^, and multiple Gaussian fitting (*R*^2^ = 0.98), based on three Gaussian functions (fit peak 1, 2, 3) centered at 0.00, 0.07, 0.49 eV, respectively. Lower panel: Relative ion yield intensity of the molecular anion radical CoQ_1_*^•^*^#–^ and multiple Gaussian fitting (*R*^2^ = 0.64) based on three Gaussian functions (fit peak 1, 2, 3) centered at 0.00, 1.10, 1.46 eV, respectively. In all panels, the solid gray line (sum) is the total fit.

The relative yield of CoQ_0_^•#–^ as a function of the incident electron energy is presented in Fig. 3. It is composed of three resonant features, dominated by the sharp ∼ 0 eV structure, followed by a shoulder at 0.07 eV, and a broad resonance centered at 0.49 eV. These results are in clear contrast to those of *p*BQ. As shown in Fig. 3, the relative *p*BQ^•#–^ yield is composed of an intense asymmetric resonance at 1.40 eV, which can result from two peaks centered at 1.28 and 1.39 eV, preceded by a peak at 0.89 eV. Details of the model fit are provided in the Supplementary Material. For the sake of completeness, we summarize the reported peak positions of the *p*BQ^•#–^ ion yield from previous mass-spectrometric experiments [22], [23], [24], [25], [47], [48] in the SI (Table S4).

If we calculate the ratio of the (pressure corrected) maximum intensities in the cumulative ion yields (for *p*BQ^•#–^ at 1.4 eV and for CoQ_0_^•#–^ at ∼ 0 eV), we obtain ∼ 11. Although the relative CoQ_0_^•#–^ yield seems therefore to be one order of magnitude lower than that of *p*BQ^•#–^ yield, we should note that the relative yield measured at ∼ 0 eV is just a lower limit. It is underestimated due to the limited electron energy resolution and limited transmission of very slow electrons in the hemispherical electron monochromator. In addition, there is a reduced transmission of heavier CoQ_0_^•#–^ ions (*m*/*z* 182) in the QMS compared to the *p*BQ^•#–^ (*m*/*z* 108), which is also the case for CoQ_1_^•#–^ (*m*/*z* 250).

Some insight into the connection between optical and EA results can be gained from the interplay between auto-ionization ($\tau_{a}$) and internal conversion ($\tau_{c}$) lifetimes. For *p*BQ, the ^2^A_u_ shape resonance has a vertical auto-ionization width around 0.01 eV, according to scattering (static plus exchange polarization approximation [32], [33]) and complex absorption potential calculations based on the EOM-CC method [35]. The auto-ionization lifetime, $\tau_{a}$, can thus be estimated as $\tau_{a}\approx 65\;\mathrm{fs}$. Since the lifetime of the ^2^A_u_ state is τ≈25 fs [15], the internal conversion lifetime, $\tau_{c}^{-1}$, can be evaluated from $\tau^{-1}\;=\;\tau_{a}^{-1}\;+\;\tau_{c}^{-1}$, such that $\tau_{c}\approx 40\;\mathrm{fs}$, in fair agreement with the TRPES results [17]. Longer auto-ionization lifetimes would be expected for the ^2^B_3u_ state, in view of the Feshbach character [15]. According to the TRPES data, at least 75 % of the photo-excited ^2^B_3u_ population decay by internal conversion, such that $\tau_{c}\;<\;40\;\;\mathrm{fs}$ and$\tau_{a}\;>\;120\;\;\mathrm{fs}$. The theoretical estimates for the auto-ionization lifetime of the ^2^B_3u_ state vary considerably. A balanced description of dynamic and static correlation among the shape and core-excited resonances of *p*BQ, as well as their coupling to the continuum, is a challenging task for computational methods. The auto-ionization widths are impacted by imprecisions in the resonance positions and also by the admixture of shape and core-excited characters in the different models. The close-coupling R-matrix calculation [32], built on CASSCF target states, is the most elaborate model reported so far. It predicts $\tau_{a}\approx 10$ fs for the ^2^B_3u_ state (vertically), which is probably underestimated since the calculated resonance position, 1.90 eV, seems overestimated. It is worth mentioning that scattering calculations [32], [33] generally predict the ^2^B_3u_ state to lie closely to much longer-lived Feshbach resonances, suggesting that prompt non-adiabatic population transfer could also suppress auto-ionization, even in case the ^2^B_3u_ state was relatively short lived. In view of the results outlined above, in particular the auto-ionization [15] and TRPES [17] data, one is lead to conclude that the observation of *p*BQ^•#–^ around 1.4 eV in EA experiments, is a consequence of the longer auto-ionization lifetimes for the ^2^B_3u_ Feshbach state (or manifold of coupled Feshbach states), compared to the internal conversion counterparts, $\tau_{a}\;>\;\;\tau_{c}$. For the ^2^A_u_ shape resonance, the absence of parent anion radical formation in EA measurements would arise from the shorter auto-ionization lifetimes and thus within the timescale for internal conversion, $\tau_{a}\approx \tau_{c}$.

TRPES measurements were also reported for CoQ_0_, along with calculations performed with the XMCQDPT2 method [37]. Although the same trend for the studied resonances in *p*BQ and CoQ_0_ was pointed out, a shorter auto-ionization lifetime was assigned to the [5]^2^F Feshbach resonance, which is the analogue of ^2^B_3u_ state in pBQ. The lack of symmetry in CoQ_0_ is expected to enhance the coupling of low partial waves, in particular *l* = 0, which tends to broaden the auto-ionization widths. Bull *et al.* [37] called attention to energetically less favorable internal conversion pathways, and also for the denser vibrational spectrum of neutral CoQ_0_, compared to that of *p*BQ. The shorter-lived Feshbach resonance(s) can therefore account for the lack of CoQ_0_^•#–^ formation around 1.4 eV (see Fig. 3a) and thus a breaking of the family rule.

We further performed CASSCF(15,12)/CASPT2 computations for the most stable CoQ_1_ conformer, as described in the SI, without including the diffuse DBS orbital in the active space. It was similar to the (13,10) space described above, although augmented with occupied (π_side_) and virtual ($\pi_{\mathrm{side}}^{*}$) orbitals located on the isoprenoid tail. Two electrons were also included, in consistency with the additional occupied orbital. The energies of the anion radical states are shown in Table 2, along with the CASSCF(13,10)/CASPT2 results for CoQ_0_. The comparison is made for the conformer B of the latter species, in view of the similar orientations of both methoxy groups.

**Table 2:** Characters and energies of the CoQ_0_ (conformer B) and CoQ_1_ anion radical states as well as the largest coefficients (in parenthesis), in units of eV, obtained from CASSCF(13,10)/CASPT2 and CASSCF(15,12)/CASPT2 calculations, respectively*.*

Anion radical state character	CoQ_0_	CoQ_1_
${\left(\pi_{1}^{*}\right)}^{1}$	–1.84	–1.58
${\left(\pi_{2}^{*}\right)}^{1}$	0.85 (0.82)	0.83 (0.82)
${\left(n_{2}\right)}^{1}{\left(\pi_{1}^{*}\right)}^{2}$	1.11 (0.87)	1.31 (0.62)
${\left(\pi_{3}^{*}\right)}^{1}$ ${\left(\pi_{3}\right)}^{1}{\left(\pi_{1}^{*}\right)}^{2}$	1.15 (0.35, 0.39)	1.22 (0.60)
		1.74 (0.72)
${\left(n_{1}\right)}^{1}{\left(\pi_{1}^{*}\right)}^{2}$	1.16 (0.88)	1.39 (0.63)
${\left(\pi_{4}\right)}^{1}{\left(\pi_{1}^{*}\right)}^{2}$	1.46 (0.70)	1.68 (0.72)
${\left(\pi_{\mathrm{side}}^{*}\right)}^{1}$		1.63 (0.62)

Table 2 indicates that there is a similarity in the energy of the resonances in CoQ_0_ and CoQ_1_, but two differences are noteworthy. The mixed-character ${\left(\pi_{3}^{*}\right)}^{1}/{\left(\pi_{3}\right)}^{1}{\left(\pi_{1}^{*}\right)}^{2}$ resonance obtained for CoQ_0_, with the CASPT2 expansion coefficients 0.35/0.39, splits into two resonances with sharper shape and Feshbach characters (coefficients of 0.60 and 0.72, respectively). Also, the $\pi_{\mathrm{side}}^{*}$ orbital gives rise to an additional shape resonance at 1.63 eV. Our calculations further suggest an additional core-excited resonance lying at 2.86 eV, which lies considerably above the energies of interest. The energy of the $\pi_{\mathrm{side}}^{*}$ state is in agreement with the estimate based on empirically corrected virtual orbital energies reported by Pshenichnyuk *et al.* [36], 1.66 eV. Employing the same method, these authors assigned shape resonances located on the side chains for the three CoQ*_n_* (*n* = 1,2,4) molecules, with positions ranging from 1.4 eV to 1.8 eV. For CoQ_1_, the energy of the $\pi_{3}^{*}$ shape resonance obtained with the CASSCF(15,12)/CASPT2 calculations, 1.22 eV, agrees with the measurements of Pshenichnyuk *et al.* [36], ≈ 1.2 eV. The present high-resolution measurement of the CoQ_1_^•#–^ parent anion radical locates the main peak at 1.1 eV with a shoulder at higher energy of ∼ 1.5 eV, see the lower panel of Fig. 3. However, for both features the nearby Feshbach states, around 1.31 eV to 1.74 eV, are expected to be mainly responsible for the stabilization of the parent anion radical. Apart from the higher density of vibrational states associated with the isoprenoid tail, our results point out that the additional $\pi_{\mathrm{side}}^{*}$ resonance could make the stabilization more efficient, as previously suggested [36]. Despite the somewhat high energy (1.74 eV), the enhanced Feshbach character of the ${\left(\pi_{3}\right)}^{1}{\left(\pi_{1}^{*}\right)}^{2}$ state in CoQ_1_, compared to CoQ_0_, could also play a part in the stabilization.

Finally, we come to the question, why we observe resonance features below ∼ 0.8 eV in CoQ_0_^•#–^ ion yield which for the other family members are either absent (*p*BQ^•#–^, upper panel of Fig. 3) or have weaker abundance than the main peak at higher energies (CoQ_1_^•#–^, lower panel of Fig. 3)? According to our theoretical results, the vertical binding energy of the $\pi_{1}^{*}$ anion radical ground state would be large enough to prevent the formation of the parent anion radical at ∼ 0 eV. Thus, the experimental CoQ_0_^•#–^ signal at ∼ 0 eV should therefore be related to the shallow DBS pointed out by the CASSCF(13,11)/CASPT2 model. The shoulder at 0.07 eV (see Fig. 3) might in turn be the signature of a vibrational progression (see below). The broad parent anion radical signal centered at 0.49 eV lies significantly below the calculated vertical energies of the excited anion radical states. While the experimental result could be viewed as compatible with the dense spectrum of low-lying shape and Feshbach resonances, the calculations do not clearly indicate why the formation of the parent around 0.49 eV is observed for CoQ_0_.

We further investigated possible explanations for theses striking differences between parent anion radical formation in CoQ_0_ and the other members of the CoQ*_n_* (*n* = 1,2,4) family in near zero-eV region. Three conformers of each neutral CoQ*_n_* molecule (*n* = 1,2,4) were optimized with the B3LYP/aug-cc-pVDZ method. The structures are shown in the SI along with their dipole moment intensities and electron affinities values as calculated at the CAM-B3LYP/aug-cc-pVDZ level. For all conformers, the $\pi_{1}^{*}$ anion radical ground states are bound by 1.53 eV to 1.72 eV, while the dipole moments range from 1.27 D to 2.08 D. Comparing the most polar conformers of each species, we find the dipole moments 2.27 D (CoQ_0_), 1.96 D (CoQ_1_), 2.08 D (CoQ_2_) and 2.06 D (CoQ_4_), according to B3LYP/aug-cc-pVDZ estimates. The deviations around 0.2 D do not seem large enough to justify the different patterns for parent anion radical formation at ∼ 0 eV. The accommodation of an extra electron into the DBS should be accompanied by vibrational excitation, which typically involves the stretch of polar bonds approximately oriented along the dipole moment direction [49]. For CoQ_0_, the DBS orbital lies close to the methoxy groups (see Fig. 2), so that the vibrational modes with significant C–O stretch character are expected to take part in the DBS formation. Six modes with such character were found for neutral CoQ_0_ with fundamental excitation energies of 0.12 eV and 0.13 eV (see the SI). In particular, a mode with O–CH_3_ stretch character, located on the hydroxyl group lying at meta position with respect to the methyl group, has a fundamental frequency of *ħω* = 0.120 eV. A simple model for electron attachment at very low energies can be obtained by assuming that the O–CH_3_ stretch mode, and possibly other close-lying vibrational modes, would have the same frequencies in the neutral ground state and in the DBS [49]. Excitation of the *υ* = 1,2 levels would thus form vibrational Feshbach resonances (VFRs) at ≈0.055 eV and ≈0.175 eV. These energies are somewhat too high compared to the experimental peaks at ≈0 eV and ≈0.07 eV, but the disagreement can be rationalized. The energy estimate for the lower-lying VFR suggests that the DBS binding energy computed with the CASSCF(13,11)/CASPT2 method would be underestimated by approximately 50 meV, apart from zero-point corrections. The higher-lying VFR, which would correlate with the shoulder at 0.07 eV in the parent anion radical signal, could be further stabilized by anharmonic effects, expected to be more important for the DBS than for the neutral form.

As mentioned above, the alignment of the dipole moment vector with the O–C3 bond (lying at meta position with respect to the methyl group, see Fig. 2) is expected to favor the formation of vibrationally excited resonances on the DBS state. For several strongly polar CoQ*_n_* (*n* = 0,1,2,4) conformers, we investigated the angle formed by the dipole moment vector with the O–C3 bond (θ_OC3_), and also with the *C*2–C3–C4 plane (θ_CCC_). The results are shown in Table 3, and the labels of the conformers are given in Fig. 1 (CoQ_0_) and Figures S3 to S5 (CoQ*_n_*, *n* = 1,2,4). Since the 6-member rings are approximately planar in all conformers, it is reasonable to consider θ_CCC_ the angle between the dipole vectors and the rings. Although the differences in the θ_OC3_ angles are modest, CoQ_0_ (B conformer) has the sharpest alignment between the dipole vector and the O–C3 bond, and thus with the v_OCH3_ stretch coordinate. The dipole vector also forms the smaller angle with the *C*2–C3–C4 plane in CoQ_0_, i.e., it has the largest dipole projection onto the approximate ring plane. The orientation of the tail seems to affect the dipole orientation in the larger CoQ*_n_* molecules, with (*n* > 0). These systems have dipole vectors more perpendicular to the approximate ring planes, and the comparison between the G and I conformers of CoQ_2_, as well as the J and K conformers of CoQ_4_, points out some variation in the θ_OC3_ and θ_CCC_ angles. While our calculations do not provide a deeper understanding of the impact of conformational changes in the measured EA yields, the formation of the CoQ_0_ parent anion radical at ∼ 0 eV could be favored by the somewhat larger dipole moment, the sharper alignment with the v_OCH3_ coordinate, and the absence of dipole vector fluctuations (magnitude and direction) induced by the conformational changes of the isoprenoid tail. These effects could explain the strongly reduced abundance in the CoQ*_n_*^•#–^ (*n* = 1,2,4) molecules at energies close to 0 eV.

**Table 3:** Dipole moment magnitudes (in Debye) along with the θ_OC3_ and θ_CCC_ angles (in degree)*.* The θ_OC3_ angle is the orientation of the dipole vector with respect to the OC3 bond, while θ_CCC_ is the angle between the dipole vector and the C2–C3–C4 plane (see text). The conformer labels (B, D, G, I, J and K) are given in Fig. 1 (CoQ_0_), S3 (CoQ_1_), S4 (CoQ_2_) and S5 (CoQ_4_).

	CoQ_0_ (B)	CoQ_1_(D)	CoQ_2_ (G)	CoQ_2_ (I)	CoQ_4_ (J)	CoQ_4_ (K)
dipole	2.27	1.96	1.97	2.08	2.06	1.93
θ_OC3_	11.5	30.5	20.1	15.8	12.9	17.8
θ_CCC_	38.6	62.1	50.4	61.8	56.2	62.3

## 5 Conclusions

The present results show that in spite of the similarity between the resonances and conical intersections in *p*BQ and CoQ_0_, the latter is a *worse* model for parent anion radical formation in the CoQ*_n_* (*n* = 1,2,4) family. This conclusion is unexpected since the electron-accepting group in biologically occurring CoQ*_n_* molecules is CoQ_0_, not *p*BQ. The comparison between our results with the recently reported EA measurements for CoQ*_n_* (*n* = 1,2,4) [36] strongly suggests that the isoprenoid tail could play a relevant role in electron transfer reactions at the mitochondrial electron transport chain. It is generally accepted that the isoprenoid side chain takes part in intermolecular interactions, although its length varies from species to species [11], [12], [13], [14]. Besides the relevance of those intermolecular interactions to the bioactivity of ubiquinone, one can consider a more direct impact of the isoprenoid side chain on the electron-accepting properties. One may assume that (i) resonances act as doorway states for electron transfer to ubiquinone, in view of the internal conversion pathways that avoid the inverted regime [17]; and (ii) hydrogen bonding or H–π interactions of the CoQ_0_ head only slightly perturbs the resonances, as observed for mono-hydrated *p*BQ [50]. According to the TRPES results [37], the stabilization of CoQ_0_, i.e., the decay to the anion radical ground state, would be inefficient at least compared to pBQ. Our EA results are compatible with enhanced auto-ionization yields at 1.4 eV and further reveal that the mechanisms for parent anion radical formation in CoQ_0_ differ from those in pBQ. Nevertheless, even the CoQ*_n_* (*n* = 1,2,4) family members efficiently stabilize the excess negative charge, according to EA measurements [36]. The energy dependence of the parent anion radical formation further suggests similar mechanisms in *p*BQ and CoQ*_n_* (*n* = 1,2,4), although not in CoQ_0_. Altogether, the experimental data point out that the isoprenoid tail plays a decisive role in the stabilization of the parent anion radicals of CoQ*_n_* (*n* = 1,2,4) species. Although the mono-hydration of *p*BQ favours the decay to the parent anion radical, according to recent TRPES measurements [51], it is unclear how hydrogen bonding would impact the resonances localized on the CoQ_0_ head. The remarkable effect of the isoprenoid tail on the CoQ*_n_*^•#–^ (*n* = 1,2,4) formation around 1.2 eV could be expected as a more efficient stabilization mechanism. The electron auto-ionization times are increased by two to three orders of magnitude, compared to *p*BQ, depending on the number of isoprenoid units forming the tail. The length of the isoprenoid tail obviously favours the electron energy redistribution, but Pshenichnyuk *et al.* [36] also called attention to additional shape resonances localized on the side chains, based on the emprical rescaling of π* virtual orbital energies. The energy proximity between the CoQ_0_ Feshbach resonances and the π* states atributted to the isoprenoid tails suggests that couplings among those anion radical states could suppress the otherwise enhanced auto-ionization channels in CoQ_0_. To that extent, the isoprenoid side chain would not be merely a spectator of electron transfer reactions mediated by ubiquinone.

## CRediT authorship contribution statement

**J. Ameixa:** Conceptualization, Formal analysis, Investigation, Writing – review & editing, Visualization. **E. Arthur-Baidoo:** Formal analysis, Investigation. **J. Pereira-da-Silva:** Investigation. **M. Ončák:** Methodology, Investigation, Writing – review & editing. **J.C. Ruivo:** Investigation. **M.T. do N. Varella:** Methodology, Investigation, Supervision, Writing – original draft. **F. Ferreira da Silva:** Supervision, Writing – original draft. **S. Denifl:** Conceptualization, Supervision, Writing – review & editing.

## Declaration of Competing Interest

The authors declare that they have no known competing financial interests or personal relationships that could have appeared to influence the work reported in this paper.
