# Supplementary material for: Parent anion radical formation in coenzyme Q0: Breaking ubiquinone family rules
Source: Comput Struct Biotechnol J. 2022 Dec 9;21:346–53. doi: 10.1016/j.csbj.2022.12.011 (PMC9792397; doi:10.1016/j.csbj.2022.12.011)
Supplement: Supplementary data 1 [file mmc1.docx]

**Supporting Information for**

**P****arent Anion Radical Formation in Coenzyme Q_0_: Breaking Ubiquinone Family Rules**

J. Ameixa^1,2,3^, E. Arthur-Baidoo^1,3^, J. Pereira-da-Silva^2^, M. Ončák^1^, J. C. Ruivo^4^, M. T. do N. Varella^4,*^, F. Ferreira da Silva^2,*^, S. Denifl^1,3,*^

^1^ Institut für Ionenphysik und Angewandte Physik, Leopold-Franzens Universität Innsbruck, Technikerstraße 25/3, 6020 Innsbruck, Austria; E-mail:stephan.denifl@uibk.ac.at

^2^ CEFITEC, Department of Physics, Universidade NOVA de Lisboa, 2829-516 Caparica, Portugal E-mail:f.ferreiradasilva@fct.unl.pt

^3^ Center for Molecular Biosciences (CMBI), Leopold-Franzens Universität Innsbruck, Technikerstraße 25/3, 6020 Innsbruck, Austria

^4^ Institute of Physics, University of São Paulo, Rua do Matão 1731, 05508-090 São Paulo, Brazil E-mail: mvarella@if.usp.br

**Table of Contents**

S-I Vertical binding energy of the π* anion state

S-II Geometry sensitivity of the resonance energies

S-III Vibrational analysis

S-IV Fitting of the experimentally obtained ion yield curves

S-V Reported peak positions of the *para*-benzoquinone parent anion radical

S-VI Larger CoQ*_n_* molecules

S-VII EOM-CCSD Calculations on systems A and B

References

Cartesian Coordinates

**S-I Vertical binding energy of the π* anion state**

The anion radical ground state of CoQ_0_ is valence-bound with π* character. The vertical binding energy (VBE), given by the difference between the ground-state energies of the anion radical and neutral forms at the optimal geometry of the latter, was calculated with the DFT and CASSCF/CASPT2 methods. In the latter case, we employed geometries optimized with the MP2/aug-cc-pVDZ method and different active spaces (see main text). The CASSCF/CASPT2 computations employed the ANO-L basis sets, although, for the conformer B, those basis sets were augmented with diffuse [7s5p] basis functions to account for the dipole bound state. The active spaces comprised 10 orbitals (2 *n*-type, 4 π -type, and 4 π*-type) and, for conformer B, a diffuse orbital with dipole-bound (DB) character was also taken into account. The number of active electrons was 12 and 13 for the neutral and anion radical species, respectively. In summary, we performed CASSCF/CASPT2 computations with (12,10) and (12,11) active spaces for the neutral form with and without the DB orbital, respectively, while (13,10) and (13,11) for the anion radical form. The DFT and MP2 calculations were carried out with the Gaussian09 package[1], while the CASSCF/CASPT2 ones with OpenMOLCAS[2]. The CASPT2 calculations employed the state-averaged CASSCF wave function as reference. We used an imaginary shift of 0.2 a.u. with no ionization potential electron-affinity (IPEA) shift as previously suggested for organic chromophores[3]. The results are shown in Tab. S1, coefficients of additional diffuse basis functions are included in Tab. S2.

**Table S1:** Vertical binding energies for the π^*^_1_ anion ground state of CoQ_0_.

**Table S2:** Coefficients of diffuse basis functions added into the basis set to describe the dipole-bound state in CASSCF/CASPT2 calculations.

| Function type | Coefficient |
| --- | --- |
| $s$ | 0.008842360 |
| $s$ | 0.002796200 |
| $s$ | 0.000888423 |
| $s$ | 0.000279620 |
| $s$ | 0.000088842 |
| $s$ | 0.000027962 |
| $s$ | 0.000008884 |
| *p* | 0.031251841 |
| *p* | 0.009882700 |
| *p* | 0.003125184 |
| *p* | 0.000988270 |
| *p* | 0.00031251 |

**S-II Geometry sensitivity of the resonance energies**

We investigated how the anion radical state energies of CoQ_0_ would vary with respect to small geometry changes. The resonance energies were calculated with the CASPT2(13,10) method (without the extra diffuse basis functions), and they are given with respect to the ground state energy of the neutral molecule obtained from CASPT2(12,10) calculations (also without the extra orbitals). The CASSCF/CASPT2 calculations were carried out with the OpenMOLCAS package[2], as described above (see also the main text). The results for the conformer C are shown in Tab. S3, where three geometries were considered, namely the MP2, DFT/B3LYP and HF optimal ground state geometries. The main differences in the geometry of the conformer C, arising from the optimization methods, are the double bond lengths, C=C and C=O, as indicated in the panel of Fig. S1. The lengths (*d*) generally follow the trend *d*_HF_ < *d*_MP2_ < *d*_B3LYP_ and clearly impact the anion radical state energies, which follow a similar trend.

Tab. S4 shows the anion radical-state energies for the three conformers computed at their respective optimal geometries calculated with the MP2/aug-cc-pVDZ method (the results for the conformers A and B appear in the main text and were reproduced here for comparison). As pointed out in the main text, the (π_3_)^1^(π_1_^*^)^2^ resonance have mixed character, with significant admixture of the (π_3_)^1^ shape resonance.

**Table S3:** Lowest-lying anion radical states of the conformer C of CoQ_0_. The main configurations and the energies (in eV) calculated with the CASPT2(13,10) method are given. The geometries of the conformer C were optimized with the Hartree-Fock (HF), MP2 and DFT/B3LYP methods with the aug-cc-pVDZ method.

| **Main configuration** | **HF** | **MP2** | **B3LYP** |
| --- | --- | --- | --- |
| $\left( \text{π}_{\text{1}}^{\text{*}} \right)^{\text{1}}$ | -1.44 | -1.84 | -1.73 |
| $\left( \text{π}_{\text{2}}^{\text{*}} \right)^{\text{1}}$ | 1.19 | 0.85 | 0.86 |
| $\left( \text{π}_{\text{3}}^{\text{*}} \right)^{\text{1}}$/${(\pi_{3})}^{\text{1}}\left( \text{π}_{\text{1}}^{\text{*}} \right)^{\text{2}}$ | 1.67 | 1.15 | 0.87 |
| ${\text{(}\text{n}_{\text{2}}\text{)}}^{\text{1}}\left( \text{π}_{\text{1}}^{\text{*}} \right)^{\text{2}}$ | 1.40 | 1.11 | 0.67 |
| ${\text{(}\text{n}_{\text{1}}\text{)}}^{\text{1}}\left( \text{π}_{\text{1}}^{\text{*}} \right)^{\text{2}}$ | 1.51 | 1.16 | 1.40 |
| ${\text{(}\text{π}_{\text{4}}\text{)}}^{\text{1}}\left( \text{π}_{\text{1}}^{\text{*}} \right)^{\text{2}}$ | 2.23 | 1.46 | 1.65 |

**Figure S1:** Structures of the conformer C optimized with the HF (left), MP2 (center) and DFT/B3LYP (right) methods using the aug-cc-pVDZ basis set. The lengths of the C=C and C=O bonds, in angstrom, are indicated in the panel.

**Table S4:** Lowest-lying anion radical states of the A, B and C conformers of CoQ_0_. The main configurations and the energies (in eV) calculated with the CASPT2(13,10) method are given. The geometries of the conformers were optimized with the MP2/aug-cc-pVDZ method.

| **Main configuration** | **A** | **B** | **C** |
| --- | --- | --- | --- |
| $\left( \text{π}_{\text{1}}^{\text{*}} \right)^{\text{1}}$ | -1.84 | -1.72 | -1.69 |
| $\left( \text{π}_{\text{2}}^{\text{*}} \right)^{\text{1}}$ | 0.85 | 1.00 | 0.96 |
| $\left( \text{π}_{\text{3}}^{\text{*}} \right)^{\text{1}}$/${(\pi_{3})}^{\text{1}}\left( \text{π}_{\text{1}}^{\text{*}} \right)^{\text{2}}$ | 1.15 | 1.27 | 1.26 |
| ${\text{(}\text{n}_{\text{2}}\text{)}}^{\text{1}}\left( \text{π}_{\text{1}}^{\text{*}} \right)^{\text{2}}$ | 1.11 | 1.30 | 1.25 |
| ${\text{(}\text{n}_{\text{1}}\text{)}}^{\text{1}}\left( \text{π}_{\text{1}}^{\text{*}} \right)^{\text{2}}$ | 1.16 | 1.39 | 1.44 |
| ${\text{(}\text{π}_{\text{4}}\text{)}}^{\text{1}}\left( \text{π}_{\text{1}}^{\text{*}} \right)^{\text{2}}$ | 1.46 | 1.58 | 1.58 |

**S-III Vibrational analysis**

The vibrational analysis was performed with the DFT/B3LYP/aug-cc-pDVZ method, using the Gaussian09[1] code. For the conformer B, the modes 26 and 28 have significant C–O stretch character, while the mode 27 C–O–C bending character. To illustrate the molecular distortion along these modes, we created animations with the Avogadro code^4^ and recorded the snapshots presented in Fig. S2.

**Figure S2:** Selected vibrational modes of the conformer B. In each plot, we show two snapshots of the mode animation. The force arrows (green) indicate that the three normal modes are delocalized, but the C–O bonds are highlighted. The fundamental excitation energies (in eV) are also given. Top: mode 26, having C–O stretch character at the *meta* position with respect to the methyl group. Center: mode 27, having C–O–C bending character at the *para* position with respect to the methyl group. Bottom: mode 28, having C–O stretch character at the *para* position with respect to the methyl group. The plots were generated with the Avogadro software package[4].

**S-IV Fitting of the experimentally obtained ion yield curves**

A nonlinear curve fit of the relative ion yields obtained experimentally for *p*BQ^•#-^, CoQ_0_^•#-^ and CoQ_1_^•#-^ was performed using OriginPro 9. For CoQ_0_^•#-^ we used the Gauss model, and for *p*BQ^•#-^ and CoQ_1_^•#-^ the Gaussian model. Although both models are interconvertible, a summary of each model fit is presented below.

Nonlinear curve fit: Gaussian model

|  | $\text{y=y}_{\text{0}}\text{+}\frac{\text{A}\text{e}^{\frac{\text{-4}\ln\text{2}{\text{(x-}\text{x}_{\text{c}}\text{)}}^{\text{2}}}{\text{w}^{\text{2}}}}}{\text{w}\sqrt{\frac{\text{π}}{\text{4}\ln\text{2}}}}$ | (1) |
| --- | --- | --- |

The equation (1) describes the full width at half maximum (FWHM) version of the Gaussian function, where *y_0_*, *A*, *x_c_*, and *w* denote the base, center, area and FWHM values.

Nonlinear curve fit: Gauss model

|  | $\text{y=y}_{\text{0}}\text{+}\frac{\text{A}}{\text{w}\sqrt{\frac{\text{π}}{\text{2}}}}\text{e}^{\text{-2}\frac{{\text{(x-}\text{x}_{\text{c}}\text{)}}^{\text{2}}}{\text{w}^{\text{2}}}}$ | (2) |
| --- | --- | --- |
| Derived parameters |  | (3) |
| Sigma: | $\text{σ=}\frac{\text{w}}{\text{2}}$ | (4) |
| Full width at half maximum: | $\text{FWHM=}\text{w}\sqrt{\text{2}\ln\text{2}}$ | (5) |
| Height: | $\text{y}_{\text{c}}\text{-} \text{y}_{\text{0}}\text{=}\frac{\text{A}}{\text{w}\sqrt{\frac{\text{π}}{\text{2}}}}$ | (6) |

The equation (2) describes the area version of the Gaussian function, where *y_0_*, *x_c_*, *w and A* denote the offset, center, width and area values. Equations (4), (5) and (6) allow to calculate further parameters values, such as the standard deviation, the FWHM and the height of the Gaussian function.

For each incident electron energy, the relative ion yields of CoQ_0_^•#-^, *p*BQ^•#-^, and CoQ_1_^•#-^ were rescaled by diving the measured ion yield intensity by the working pressure value of 6.0E-8 mbar, 2.6E–8 mbar and 1E–8 mbar, respectively. Tables S5, S6 and S7 summarize the multiple Gaussian fit parameters of the relative ion yields of CoQ_0_^•#-^ and *p*BQ^•#-^ before rescaling according to the working pressure value.

**Table S5:** Summary of the multiple gaussian fit of the relative ion yield of CoQ_0_^•#-^ presented in Fig.3.

|  | | **Peak 1** | **Peak 2** | **Peak 3** |
| --- | --- | --- | --- | --- |
| ***y_0_*** | Value | 0 | 0 | 0 |
|  | Standard error | 0 | 0 | 0 |
| ***x_c_*** | Value | -0.00408 | 0.07057 | 0.49043 |
|  | Standard error | 0.00116 | 0.00743 | 0.0032 |
| ***w*** | Value | 0.06846 | 0.14994 | 0.35206 |
|  | Standard error | 0.00388 | 0.00756 | 0.00715 |
| ***A*** | Value | 0.23644 | 0.36836 | 0.83886 |
|  | Standard error | 0.02968 | 0.03344 | 0.01379 |
| **sigma, *σ, eq. (3)*** | Value | 0.03423 | 0.07497 | 0.17603 |
| **FWHM, eq. (4)** | Value | 0.08061 | 0.17654 | 0.41452 |
| **Height, (*y_c_- y_0_), eq. (5)*** | Value | 2.75567 | 1.9602 | 1.90113 |
| **Statistics** | Reduced Chi-Sqr | 0.01405 | | |
|  | Adj. R-Square | 0.9841 | | |

**Table S6:** Summary of the multiple gaussian fit of the relative ion yield of pBq^•#-^ presented in Fig. 3.

|  |  | Peak 1 | Peak 2 | Peak 3 |
| --- | --- | --- | --- | --- |
| ***y_0_*** | Value | 0 | 0 | 0 |
|  | Standard error | 0 | 0 | 0 |
| ***x_c_*** | Value | 0.90775 | 1.28089 | 1.44766 |
|  | Standard error | 0.01857 | 0.81123 | 0.23418 |
| ***A*** | Value | 0.20411 | 3.76903 | 4.83267 |
|  | Standard error | 0.15973 | 25.717 | 25.6618 |
| ***w*** | Value | 0.1017 | 0.36831 | 0.31964 |
|  | Standard error | 0.054 | 0.54685 | 0.16101 |
| **Statistics** | Reduced Chi-Sqr | 0.42116 | | |
|  | Adj. R-Square | 0.99053 | | |

**Table S7:** Summary of the multiple gaussian fit of the relative ion yield of CoQ_1_^•#-^presented in Fig. 3.

|  |  | Peak 1 | Peak 2 | Peak 3 |
| --- | --- | --- | --- | --- |
| ***y_0_*** | Value | 0 | 0 | 0 |
|  | Standard error | 0 | 0 | 0 |
| ***x_c_*** | Value | 0.01463 | 1.09825 | 1.46495 |
|  | Standard error | 010505 | 0.27873 | 0.71635 |
| ***A*** | Value | 0.04489 | 0.47762 | 0.0991 |
|  | Standard error | 0.00984 | 0.57515 | 0.57385 |
| ***w*** | Value | 0.14008 | 0.60981 | 0.52512 |
|  | Standard error | 0.03545 | 0.24043 | 0.56687 |
| **Statistics** | Reduced Chi-Sqr | 0.03058 | | |
|  | Adj. R-Square | 0.64675 | | |

**S-V Reported peak positions of the *para*-benzoquinone parent anion radical**

**Table S8**: Peak positions unambiguously related to long-lived *p*BQ^•#–^ detection reported by gas-phase studies. The qualitative best agreement with present data is found with Gordon et al., where the electron beam produced by a trochoidal electron monochromator had an energy resolution of 50 meV, and a QMS was also used for the detection of anion radicals.

|  | *p*BQ^•#–^ peak positions (eV) | | | |
| --- | --- | --- | --- | --- |
|  | 1. | 2. | 3. | 4. |
| This work | 0.91±0.02 | 1.28±0.82 | 1.45±0.23 | 1.71±0.09 |
| Christophorou *et al.*[5] *^(1)^* | 2.1 |  |  |  |
| Collins *et al.* [6] *^(1)^* | 2.1 |  |  |  |
| Gordon *et al.* [7]*^(2)^* | 0.8 | 1.0 | 1.3 |  |
| Cooper *et al.* [8]*^(3)^* | 1.4±0.1 |  |  |  |
| Asfandiarov *et al.*[9] ^(4)^ | 1.35 at 348 K 1.19 at 533 K |  |  |  |
| Khvostenko *et al.*[10] *^(4)^* | 1.36 | 1.56 |  |  |

1. Electron swarm experiments;
2. Detection of *p*BQ anion radicals resulting from electron attachment with a QMS;
3. SF_6_ scaveging experiment;
4. Detection of *p*BQ anion radicals resulting from electron attachment with a magnetic sector mass spectrometer.

**S-VI Larger CoQ*_n_* molecules**

The geometries of three conformers were optimized for the neutral CoQ_1_, CoQ_2_ and CoQ_4_ molecules, labelled D to L, employing the DFT/B3LYP/aug-cc-pVDZ method. The electron affinities were obtained at the DFT/CAM-B3LYP/aug-cc-pVDZ level. The structures, relative energies, electron affinities and dipole moments are shown in Figs. S3 to S5.

The most strongly polar CoQ*n* species (*n* = 0,1,2,4) always have dipole moment vectors reasonably aligned with the O–CH_3_ bonds. This is illustrated for CoQ_1_ and CoQ_2_ in Fig. S6, where the electronic densities are also shown. The tail also seems to affect the orientation of the dipole with respect to the 6-member ring.


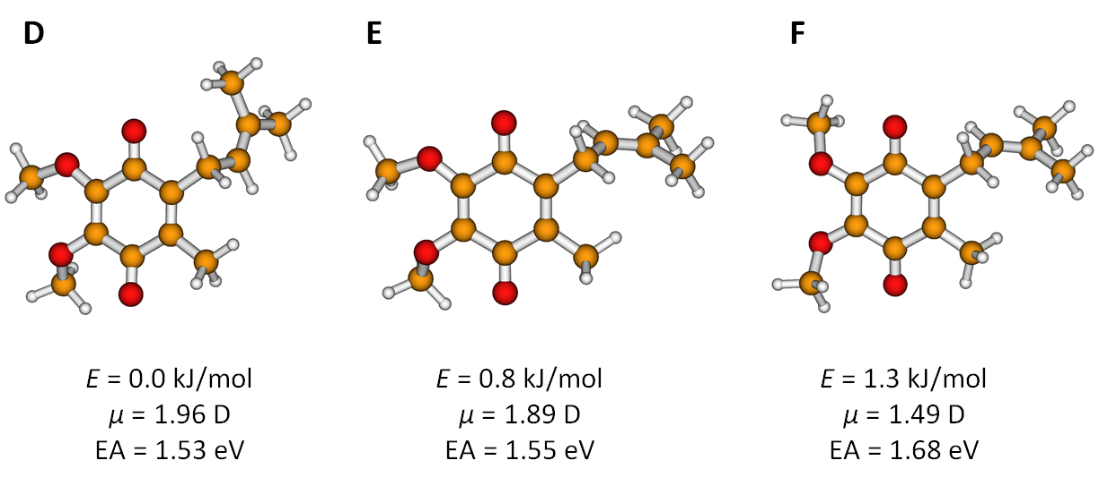


**Figure S3:** Conformers D, E and F of the CoQ_1_ molecule. Relative energies *E*, dipole moments *µ* and vertical electronic affinities EA are indicated in the panels. Optimized at the B3LYP/aug-cc-pVDZ level, electronic affinities were calculated at the CAM-B3LYP/aug-cc-pVDZ level.


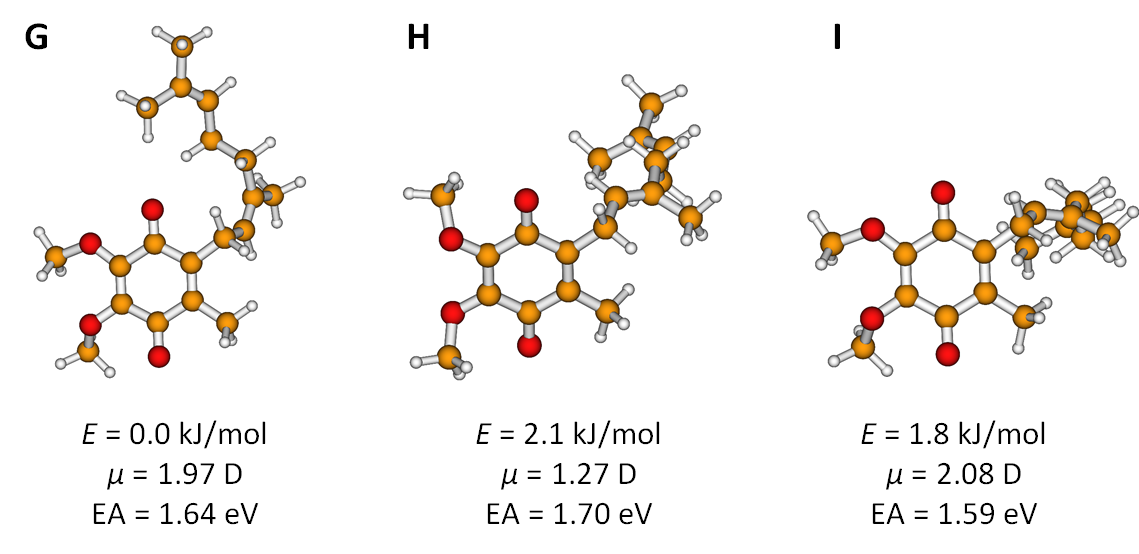


**Figure S4:** Conformers G, H and I of the CoQ_2_ molecule. Relative energies *E*, dipole moments *µ* and vertical electronic affinities EA are indicated in the panels. Optimized at the B3LYP/aug-cc-pVDZ level, electronic affinities were calculated at the CAM-B3LYP/aug-cc-pVDZ level.


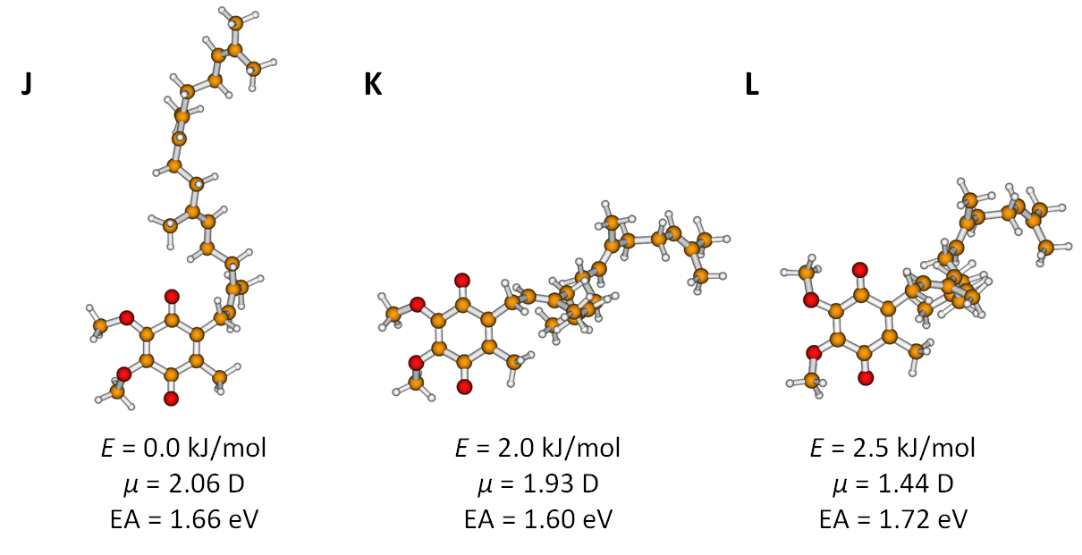


**Figure S5:** Conformers J, K and L of the CoQ_4_ molecule. Relative energies *E*, dipole moments *µ* and vertical electronic affinities EA are indicated in the panels. Optimized at the B3LYP/aug-cc-pVDZ level, electronic affinities were calculated at the CAM-B3LYP/aug-cc-pVDZ level.

**Figure S6:** Most strongly polar conformers of CoQ_1_ (D, left) and CoQ_2_ (I, right). The dipole moment vectors are indicated along with the electronic density. The latter is shown as color map where the negative and positive charges are indicated in red and blue, respectively.

**S-VII EOM-CCSD Calculations on systems A and B**

**Table S9:** EOM-CCSD calculations on systems A and B. See Table 1 for further details. For states given in parenthesis, the character could not be determined unequivocally.

|  | EOM-CCSD/  6-31+g* | |
| --- | --- | --- |
| Anion radical state character | A | B |
| ${\text{(}\text{π}_{\text{1}}^{\text{*}}\text{)}}^{\text{1}}$ | -1.44 | –1.18 |
| ${\text{(}\text{π}_{\text{2}}^{\text{*}}\text{)}}^{\text{1}}$ | (1.46) | (1.71) |
| ${\text{(}\text{n}_{\text{2}}\text{)}}^{\text{1}}{\text{(}\text{π}_{\text{1}}^{\text{*}}\text{)}}^{\text{2}}$ | (1.65) | (1.93) |
| ${\text{(}\text{n}_{\text{1}}\text{)}}^{\text{1}}{\text{(}\text{π}_{\text{1}}^{\text{*}}\text{)}}^{\text{2}}$ | (1.70) | (2.23) |

**References**

[1] M. J. Frisch, G. W. Trucks, H. B. Schlegel, G. E. Scuseria, M. A Robb, J. R. Cheeseman, G. Scalmani, V. Barone, B. Mennucci, G. A. Petersson et al., Gaussian 09, revision D.01, Gaussian, Inc., Wallingford, CT, 2009.

[2] I. Fdez. Galván, M. Vacher, A. Alavi, C. Angeli, F. Aquilante, J. Autschbach, J. J. Bao, S. I. Bokarev, N. A. Bogdanov, R. K. Carlson, et al., *J. Chem. Theory Comput.* **2019**, *15*, *5925*–*5964*.

[3] J. P. Zobel, J. J. Nogueira, L. González, *Chem. Sci.* **2017**, *8*, *1482*–*1499*.

[4] M. D. Hanwell, et al., *J. Cheminform*. **2012**, *4*, *1*–*17* (2012).

[5] L. G. Christophorou, J. G. Carter, A. A. Christodoulides, *Chem. Phys. Lett.* **1969**, *3*, 237–240.

[6] P. M. Collins, L. G. Christophorou, E. L. Chaney, J. G. Carter, *Chem. Phys. Lett.* **1970**, *4*, 646–650.

[7] R. L. Gordon, D. R. Sieglaff, G. H. Rutherford, K. L. Stricklett, *Int. J. Mass Spectrom. Ion Process.* **1997**, *164*, 177–191.

[8] C. D. Cooper, W. T. Naffs, R. N. Compton, *J. Chem. Phys.* **1975**, *63*, 2752–2757.

[9] N. L. Asfandiarov, S. A. Pshenichnyuk, A. I. Fokin, E. P. Nafikova, *Chem. Phys.* **2004**, *298*, 263–266.

[10] O. G. Khvostenko, P. V. Shchukin, G. M. Tuimedov, M. V. Muftakhov, E. E. Tseplin, S. N. Tseplina, V. A. Mazunov, *Int. J. Mass Spectrom.* **2008**, *273*, 69–77.

**Cartesian Coordinates (in Å) for structures optimized at the B3LYP/aug-cc-pVDZ level along with electronic energies including zero-point correction (in Hartree)**

CoQ_1_

E = -844.965161

C -0.142254 1.637453 -0.462060

C -1.594834 1.440947 -0.145343

C -2.156666 0.071968 -0.033983

C -1.370570 -1.008162 -0.312942

C 0.067053 -0.806889 -0.719501

C 0.644463 0.573065 -0.749447

C 0.313864 3.069570 -0.444073

C -4.014986 0.447712 1.432702

C -2.907840 -2.793436 0.276907

C 2.120489 0.654671 -1.083727

C 2.973339 0.678350 0.167166

C 3.873677 -0.235205 0.569682

C 4.205024 -1.505267 -0.171878

O -2.320993 2.420938 0.006898

O -3.499369 -0.058737 0.186974

O -1.696920 -2.307683 -0.324069

O 0.747319 -1.777959 -1.013277

H 0.176564 3.499171 0.558465

H -0.305670 3.675605 -1.118181

H 1.364467 3.173906 -0.726843

H -5.082588 0.204716 1.428018

H -3.875337 1.530589 1.505020

H -3.525779 -0.060458 2.278408

H -2.809558 -3.882935 0.248447

H -3.788941 -2.472546 -0.286982

H -2.984927 -2.454838 1.318566

H 2.379469 -0.198288 -1.717000

H 2.308740 1.565215 -1.670054

H 2.820237 1.553609 0.805782

H 3.544631 -1.694656 -1.022669

H 4.129770 -2.369244 0.506500

H 5.246648 -1.478716 -0.530564

C 4.651404 -0.030544 1.847572

H 4.390168 0.914794 2.339669

H 5.735409 -0.029689 1.649605

H 4.463421 -0.854110 2.555148

E = -844.964839

C 0.230066 1.172584 -0.482634

C -1.213343 1.475236 -0.209111

C -2.192770 0.370799 -0.057794

C -1.791669 -0.921006 -0.239343

C -0.353401 -1.221388 -0.575404

C 0.630282 -0.102672 -0.698791

C 1.131290 2.374147 -0.521440

C -3.875026 1.419882 1.291387

C -3.832690 -2.074711 0.391897

C 2.049980 -0.514390 -1.038873

C 2.868834 -0.800306 0.200638

C 4.080389 -0.315489 0.522725

C 4.883065 0.642085 -0.322550

O -1.583835 2.644904 -0.135780

O -3.511425 0.693129 0.102171

O -2.522047 -2.043403 -0.195365

O -0.000397 -2.381919 -0.722721

H 2.185790 2.093529 -0.569676

H 0.969185 2.994960 0.369038

H 0.886583 3.011680 -1.383203

H -4.963594 1.528676 1.246323

H -3.398114 2.404424 1.311946

H -3.600074 0.840705 2.186759

H -4.088887 -3.137749 0.433731

H -4.556873 -1.528924 -0.220468

H -3.810144 -1.658183 1.407650

H 1.991882 -1.432027 -1.641060

H 2.517791 0.255910 -1.659814

H 2.402600 -1.505523 0.893914

H 4.372628 0.947358 -1.242152

H 5.843059 0.185553 -0.610763

H 5.130706 1.549888 0.250469

C 4.756034 -0.730640 1.807850

H 5.726959 -1.210603 1.604582

H 4.139480 -1.432281 2.383198

H 4.966406 0.146146 2.441752

E = -844.964647

C 2.265751 -0.356427 0.171007

C 1.309793 -1.495626 0.134112

C -0.099941 -1.264578 -0.325865

C -0.510007 -0.016546 -0.649067

C 0.441438 1.137997 -0.573053

C 1.829056 0.913935 -0.087643

O 1.663227 -2.609418 0.512348

C -0.998111 -2.471663 -0.375426

O 0.086689 2.254406 -0.944196

O 2.718820 1.927348 -0.029253

C 2.319498 3.222349 0.463113

O 3.534844 -0.528549 0.597039

C 4.293351 -1.683793 0.185959

C -1.916761 0.309117 -1.105762

H -0.523507 -3.318482 0.128464

H -1.962557 -2.260076 0.102431

H -1.200903 -2.767220 -1.416185

H 5.341045 -1.372107 0.256205

H 4.097283 -2.536058 0.842821

H 4.060165 -1.954710 -0.851851

H 3.244625 3.682390 0.826623

H 1.873389 3.824927 -0.333134

H 1.609211 3.121474 1.293899

H -1.854889 1.158221 -1.801191

H -2.328717 -0.543041 -1.655944

C -2.806699 0.696888 0.055497

C -4.009310 0.193236 0.382813

H -2.406846 1.501415 0.678760

C -4.764835 0.725739 1.576938

C -4.727125 -0.896013 -0.374803

H -4.981239 -1.731073 0.297157

H -5.681431 -0.518261 -0.774544

H -4.149698 -1.297439 -1.214037

H -4.967135 -0.077758 2.303602

H -4.209215 1.521399 2.088475

H -5.744818 1.128407 1.274024

CoQ_2_

E = -1040.206457

C 0.950300 -1.025756 -0.759606

C 2.193810 -1.562921 -0.757436

C 3.389227 -0.704630 -0.466685

C 3.211156 0.712598 -0.064004

C 1.961178 1.260001 -0.049060

C 0.768463 0.420959 -0.428277

C 2.514364 -3.001570 -1.051724

O 4.517768 -1.177831 -0.579740

O 4.327802 1.478032 0.121231

C 5.222810 1.096608 1.183665

O 1.596720 2.519073 0.229607

C 2.480944 3.436600 0.893233

O -0.340115 0.934632 -0.450332

C -0.319950 -1.798715 -1.051073

C -0.848233 -2.488570 0.190071

C -2.090589 -2.425792 0.700585

C -2.436769 -3.198220 1.952108

C -3.222819 -1.617543 0.104094

H 3.029755 -3.457769 -0.194853

H 3.214664 -3.071385 -1.894783

H 1.620451 -3.589120 -1.275137

H 6.008816 1.858753 1.191373

H 5.656377 0.109478 0.997332

H 4.690570 1.107317 2.147603

H 1.852567 4.301080 1.128168

H 3.310911 3.730365 0.243652

H 2.868093 2.996046 1.821513

H -1.061044 -1.102404 -1.452424

H -0.112971 -2.546000 -1.831276

H -0.114591 -3.109267 0.713167

H -3.037854 -1.394715 -0.953744

C -3.488093 -0.281455 0.845963

H -4.141810 -2.225215 0.136325

H -1.568416 -3.737187 2.351208

H -3.234804 -3.929713 1.748058

H -2.817192 -2.531191 2.740911

C -4.738502 0.396687 0.354386

H -3.598088 -0.489762 1.922507

H -2.606887 0.360028 0.736248

C -4.851396 1.566089 -0.300420

H -5.665096 -0.152809 0.556509

C -6.206397 2.088782 -0.714499

C -3.693866 2.458398 -0.674255

H -3.714562 2.672805 -1.754851

H -3.781419 3.432174 -0.165340

H -2.715241 2.033085 -0.433913

H -6.400706 3.076243 -0.264459

H -6.257549 2.228798 -1.806699

H -7.015203 1.409817 -0.415630

E = -1040.205640

C 3.436923 -0.573199 -0.564304

C 2.684926 0.388260 -1.414538

C 1.640033 1.267489 -0.792161

C 1.348386 1.144137 0.523138

C 2.077724 0.147716 1.371435

C 3.089267 -0.745988 0.747442

O 2.893164 0.435304 -2.624120

C 0.951248 2.243309 -1.708368

O 1.866666 0.096290 2.580841

O 3.780402 -1.639178 1.486700

C 3.114863 -2.419453 2.500149

O 4.369201 -1.390371 -1.098185

C 5.307081 -0.890122 -2.072364

C 0.299399 1.977162 1.229903

H 1.156068 1.991245 -2.752803

H -0.132145 2.240946 -1.539845

H 1.313698 3.267982 -1.533270

H 6.177540 -1.549806 -1.989689

H 4.884610 -0.929786 -3.080426

H 5.603742 0.139765 -1.835357

H 3.729405 -3.319283 2.610807

H 3.057566 -1.870286 3.444201

H 2.105643 -2.702489 2.174263

H 0.626713 2.115439 2.270169

H 0.242753 2.965488 0.762967

C -1.048726 1.288573 1.232581

C -2.235756 1.778128 0.832253

H -1.030399 0.271223 1.633029

C -3.477329 0.913603 0.923315

C -2.452390 3.173543 0.298605

H -2.883049 3.152678 -0.713648

H -3.172801 3.712862 0.933138

H -1.533834 3.768018 0.259891

C -4.024495 0.452946 -0.451218

H -3.262752 0.022223 1.528200

H -4.273173 1.469459 1.447111

C -5.287414 -0.353650 -0.315613

H -4.236987 1.339545 -1.069712

H -3.239395 -0.109618 -0.972100

C -5.491755 -1.646979 -0.622234

H -6.136907 0.199123 0.100686

C -6.841563 -2.288482 -0.406968

C -4.446986 -2.570922 -1.197853

H -4.320544 -3.455229 -0.552844

H -4.765819 -2.947842 -2.182943

H -3.467313 -2.096978 -1.317421

H -6.764400 -3.148172 0.278536

H -7.567523 -1.579344 0.010381

H -7.246770 -2.679937 -1.354399

E = -1040.205760

C 3.430909 0.476152 0.390507

C 2.693347 -0.307593 1.413576

C 1.562718 -1.203847 1.002373

C 1.275532 -1.374595 -0.309406

C 2.029473 -0.615677 -1.349632

C 3.126554 0.329580 -0.932004

O 3.025441 -0.240840 2.595024

C 0.822352 -1.902020 2.111008

O 1.754528 -0.736851 -2.534211

O 3.700614 0.903447 -1.997851

C 4.565564 2.043857 -1.875685

O 4.509405 1.211394 0.797521

C 4.259899 2.303099 1.702882

C 0.185749 -2.296307 -0.817782

H 1.028570 -1.418365 3.070132

H -0.257415 -1.899707 1.922069

H 1.145329 -2.951066 2.196541

H 5.233359 2.777207 1.865496

H 3.570711 3.027225 1.240756

H 3.853812 1.941826 2.652589

H 4.735354 2.370270 -2.906232

H 4.071132 2.844351 -1.309652

H 5.512247 1.776413 -1.396606

H 0.498260 -2.673995 -1.801763

H 0.091534 -3.154730 -0.145029

C -1.132448 -1.569318 -0.975999

C -2.335067 -1.906775 -0.476996

H -1.074325 -0.670820 -1.596124

C -3.541109 -1.034231 -0.762589

C -2.604654 -3.136042 0.356810

H -3.025977 -2.870206 1.337895

H -3.351706 -3.773742 -0.140935

H -1.711129 -3.744596 0.529810

C -4.057876 -0.252960 0.471770

H -3.295173 -0.312129 -1.552807

H -4.363246 -1.660366 -1.148281

C -5.288612 0.553177 0.156577

H -4.300427 -0.967274 1.274703

H -3.246397 0.380183 0.852425

C -5.436937 1.889751 0.156994

H -6.164358 -0.044293 -0.120119

C -6.761716 2.520928 -0.198543

C -4.348712 2.876437 0.501714

H -4.193858 3.581887 -0.330384

H -4.640185 3.483402 1.373993

H -3.387939 2.401267 0.724907

H -6.657373 3.194506 -1.064778

H -7.520849 1.765980 -0.439005

H -7.139667 3.137880 0.633101

CoQ_4_

E = -1430.688559

C -4.662654 1.022606 -0.826689

C -6.000172 1.211981 -0.926727

C -6.941206 0.050880 -0.797763

C -6.423885 -1.294432 -0.444126

C -5.078858 -1.493084 -0.326868

C -4.127866 -0.344700 -0.543513

C -6.669240 2.532435 -1.187089

O -8.141446 0.217069 -1.002843

O -7.305369 -2.337885 -0.408399

C -8.356700 -2.282454 0.574906

O -4.414455 -2.628160 -0.070123

C -5.073116 -3.791725 0.455657

O -2.924338 -0.544956 -0.474411

C -3.625977 2.120469 -0.952744

C -3.407243 2.833054 0.366119

C -2.242443 3.050985 1.001708

C -2.223424 3.798473 2.314709

C -0.890504 2.598561 0.493443

H -7.357129 2.777051 -0.365499

H -7.290334 2.473483 -2.090827

H -5.950329 3.348308 -1.295422

H -8.907963 -3.222191 0.466638

H -9.021030 -1.432358 0.392908

H -7.924793 -2.223701 1.586134

H -4.258036 -4.474794 0.713337

H -5.733839 -4.245959 -0.288778

H -5.643439 -3.537122 1.358730

H -2.694906 1.673592 -1.311193

H -3.959514 2.842156 -1.712822

H -4.320465 3.210815 0.835676

H -0.920573 2.388400 -0.582378

C -0.342476 1.343686 1.220916

H -0.171756 3.423433 0.627985

H -3.233782 4.078473 2.637965

H -1.620026 4.716218 2.229985

H -1.764982 3.194271 3.112496

C 1.072908 1.032217 0.815265

H -0.367125 1.524031 2.307882

H -1.015943 0.502095 1.025578

C 1.541127 -0.063363 0.189863

H 1.799890 1.814919 1.061092

C 3.018498 -0.184979 -0.122682

C 0.683318 -1.231916 -0.234706

H 0.935075 -1.532542 -1.263629

H 0.867496 -2.111375 0.401827

H -0.388082 -1.014665 -0.200397

C 3.733030 -1.321699 0.650239

H 3.148618 -0.367069 -1.203419

H 3.524615 0.763726 0.103939

C 5.189487 -1.422419 0.287896

H 3.592616 -1.155614 1.725924

H 3.241863 -2.278451 0.411730

C 6.268746 -1.235695 1.069598

H 5.376898 -1.670896 -0.762914

C 7.661484 -1.366199 0.486693

C 6.208083 -0.898071 2.540371

H 6.808511 -1.615030 3.122413

H 6.630564 0.098915 2.738697

H 5.188399 -0.911001 2.938325

C 8.447675 -0.032823 0.422118

H 8.245177 -2.083737 1.088829

H 7.598160 -1.782040 -0.528327

C 9.834130 -0.219139 -0.131418

H 7.869080 0.688656 -0.168871

H 8.527350 0.386554 1.437755

C 10.359649 0.280525 -1.263988

H 10.482637 -0.860373 0.476140

C 11.783918 -0.029458 -1.658429

C 9.625474 1.175390 -2.231958

H 10.137787 2.146674 -2.324410

H 9.621350 0.729019 -3.239445

H 8.587009 1.366165 -1.942461

H 11.821623 -0.525689 -2.642060

H 12.376932 0.895108 -1.752351

H 12.276695 -0.680642 -0.925378

E = -1430.687792

C -6.842771 -1.040087 0.486414

C -6.518852 0.249745 1.146896

C -5.617379 1.236999 0.466156

C -5.176738 0.997965 -0.791233

C -5.519848 -0.281666 -1.477966

C -6.376632 -1.299459 -0.770006

O -7.009488 0.519632 2.240963

C -5.282380 2.483232 1.240265

O -5.097850 -0.522010 -2.599424

O -6.594398 -2.370244 -1.545236

C -7.124188 -3.597017 -1.017976

O -7.737296 -1.871448 1.101060

C -7.358882 -2.420811 2.377360

C -4.306115 1.959629 -1.574120

H -5.486946 2.336662 2.304774

H -4.230737 2.759159 1.101985

H -5.899207 3.330516 0.902827

H -8.179813 -3.085799 2.664993

H -6.430501 -3.004697 2.277132

H -7.234457 -1.631035 3.124443

H -7.027394 -4.312763 -1.840019

H -6.530260 -3.936086 -0.158925

H -8.173144 -3.485639 -0.727217

H -4.545104 1.835818 -2.640023

H -4.555195 2.987934 -1.293308

C -2.833844 1.671472 -1.372669

C -1.861588 2.523530 -1.001392

H -2.549214 0.636954 -1.582245

C -0.436218 2.026449 -0.864596

C -2.068503 3.991980 -0.718780

H -1.764869 4.249092 0.307068

H -1.441691 4.598387 -1.390943

H -3.106188 4.314731 -0.851756

C 0.074315 1.972567 0.597594

H -0.353588 1.021068 -1.298963

H 0.236588 2.680327 -1.444728

C 1.507054 1.520040 0.676324

H -0.004621 2.976909 1.043542

H -0.589772 1.318171 1.175977

C 2.008701 0.406877 1.241794

H 2.221155 2.194075 0.190102

C 3.497302 0.130011 1.184042

C 1.181402 -0.631058 1.962127

H 1.201632 -1.594949 1.431057

H 1.591960 -0.817094 2.967007

H 0.133010 -0.337209 2.075526

C 3.878435 -1.097725 0.319227

H 4.020195 1.011317 0.787889

H 3.878869 -0.034227 2.206466

C 5.363166 -1.340212 0.309204

H 3.376736 -1.991147 0.724186

H 3.485015 -0.947839 -0.694083

C 6.215671 -1.307036 -0.731371

H 5.791254 -1.561623 1.293430

C 7.693022 -1.563182 -0.512066

C 5.808245 -1.030679 -2.159099

H 6.174940 -1.829737 -2.822515

H 4.723423 -0.958831 -2.286531

H 6.249966 -0.091316 -2.525358

C 8.593099 -0.327174 -0.760600

H 7.857309 -1.912667 0.516553

H 8.026833 -2.374863 -1.181257

C 10.050687 -0.638637 -0.556189

H 8.448648 0.019587 -1.796318

H 8.258265 0.489595 -0.108375

C 10.899890 -0.123267 0.350297

H 10.452327 -1.396181 -1.238594

C 12.340445 -0.572903 0.403724

C 10.535584 0.923774 1.373890

H 11.146183 1.830176 1.232617

H 9.481293 1.216672 1.337276

H 10.752424 0.558326 2.390594

H 12.585366 -0.994912 1.392191

H 12.558450 -1.331979 -0.358146

H 13.024637 0.277547 0.249255

E = -1430.687591

C -6.848682 -0.994665 0.803881

C -6.463193 0.371013 1.250377

C -5.639932 1.241064 0.346261

C -5.205010 0.761416 -0.841614

C -5.558129 -0.628987 -1.273411

C -6.347432 -1.501233 -0.363813

O -6.786468 0.768743 2.366580

C -5.326116 2.624956 0.848898

O -5.228486 -1.030869 -2.386830

O -6.702912 -2.750411 -0.731622

C -5.759421 -3.610166 -1.402144

O -7.573695 -1.807083 1.601401

C -8.695770 -1.289941 2.344684

C -4.357279 1.562225 -1.808006

H -5.565932 2.704708 1.913006

H -4.267324 2.866101 0.696564

H -5.920173 3.380765 0.312524

H -9.347456 -2.154897 2.508576

H -8.371690 -0.862295 3.297743

H -9.233425 -0.530927 1.761904

H -6.100178 -4.627480 -1.181830

H -5.757822 -3.426456 -2.480261

H -4.748005 -3.467293 -1.000238

H -4.614883 1.239053 -2.826635

H -4.608176 2.624010 -1.720478

C -2.879767 1.322535 -1.582555

C -1.908508 2.231111 -1.382392

H -2.589059 0.268752 -1.608592

C -0.478279 1.772912 -1.177785

C -2.121731 3.725389 -1.361523

H -1.790882 4.163161 -0.407894

H -1.520129 4.203985 -2.149870

H -3.165923 4.014593 -1.518496

C 0.038986 1.959045 0.271324

H -0.389008 0.711162 -1.444509

H 0.186150 2.329497 -1.859895

C 1.478453 1.544885 0.413648

H -0.052906 3.020167 0.552760

H -0.613664 1.396464 0.950491

C 1.994339 0.526320 1.125705

H 2.184457 2.158609 -0.156919

C 3.487629 0.269092 1.111218

C 1.179873 -0.418535 1.976558

H 1.569724 -0.438192 3.006427

H 0.120338 -0.147288 2.022022

H 1.243409 -1.449811 1.597361

C 3.895565 -1.043343 0.395541

H 4.000913 1.107168 0.620046

H 3.862251 0.229732 2.148418

C 5.382462 -1.268794 0.436685

H 3.395738 -1.891739 0.889614

H 3.518103 -1.012008 -0.634317

C 6.255514 -1.323540 -0.585790

H 5.792864 -1.390969 1.445427

C 7.730548 -1.540537 -0.314746

C 5.874555 -1.187367 -2.040773

H 6.259174 -2.042806 -2.618184

H 4.792014 -1.136236 -2.195096

H 6.317743 -0.283529 -2.486131

C 8.622524 -0.320609 -0.655330

H 7.876762 -1.793947 0.744282

H 8.087061 -2.404924 -0.901066

C 10.078871 -0.594624 -0.395651

H 8.495727 -0.069323 -1.720484

H 8.265722 0.546860 -0.085357

C 10.901693 0.004778 0.483092

H 10.504202 -1.400446 -1.004397

C 12.345803 -0.419597 0.603984

C 10.502288 1.127168 1.409049

H 11.104866 2.027312 1.206590

H 9.445564 1.401254 1.326255

H 10.700894 0.849337 2.456714

H 13.023335 0.424479 0.394879

H 12.573370 -0.756010 1.628764

H 12.589617 -1.235463 -0.0881
